# Stromal thrombospondin 1 suppresses angiogenesis in oral submucous fibrosis

**DOI:** 10.1038/s41368-024-00286-z

**Published:** 2024-02-26

**Authors:** Xiao Yang, Hui Zhao, Rui Li, Yang Chen, Zhi Xu, Zhengjun Shang

**Affiliations:** 1 State Key Laboratory of Oral & Maxillofacial Reconstruction and Regeneration, Key Laboratory of Oral Biomedicine Ministry of Education, Hubei Key Laboratory of Stomatology, School & Hospital of Stomatology, Wuhan University, Wuhan, China; 2 Department of Oral and Maxillofacial-Head and Neck Oncology, School of Stomatology–Hospital of Stomatology, Wuhan University, Wuhan, China; 3 Department of Stomatology, Union Hospital, Tongji Medical College, Huazhong University of Science and Technology, Wuhan, China

**Keywords:** Mechanisms of disease, Lab-on-a-chip

## Abstract

A decline in mucosal vascularity is a histological hallmark of oral submucous fibrosis (OSF), a premalignant disease that is largely induced by betel quid chewing. However, the lack of available models has challenged studies of angiogenesis in OSF. Here, we found that the expression of thrombospondin 1 (THBS1), an endogenous angiostatic protein, was elevated in the stroma of tissues with OSF. Using a fibroblast-attached organoid (FAO) model, the overexpression of THBS1 in OSF was stably recapitulated in vitro. In the FAO model, treatment with arecoline, a major pathogenic component in areca nuts, enhanced the secretion of transforming growth factor (TGF)-β1 by epithelial cells, which then promoted the expression of THBS1 in fibroblasts. Furthermore, human umbilical vein endothelial cells (HUVECs) were incorporated into the FAO to mimic the vascularized component. Overexpression of THBS1 in fibroblasts drastically suppressed the sprouting ability of endothelial cells in vascularized FAOs (vFAOs). Consistently, treatment with arecoline reduced the expression of CD31 in vFAOs, and this effect was attenuated when the endothelial cells were preincubated with neutralizing antibody of CD36, a receptor of THBS1. Finally, in an arecoline-induced rat OSF model, THBS1 inhibition alleviated collagen deposition and the decline in vascularity in vivo. Overall, we exploited an assembled organoid model to study OSF pathogenesis and provide a rationale for targeting THBS1.

## Introduction

Oral submucous fibrosis (OSF) is largely induced by addictive betel quid chewing^1–3^. Progressive OSF can lead to rigidity of the oral mucosa, limited mouth opening, and masticatory damage^4,5^. In addition, OSF appears to be the most common pathology that can harbour a second pathogenically distinct precancerous lesion in the oral cavity, which may increase the malignant transformation rate of oral squamous cell carcinoma (OSCC)^3,6^. Therefore, OSF is a typical premalignant disease, and in-depth study of OSF is important for protecting oral health.

A change in mucosal vascularity is a histological hallmark of OSF. In mucosa with early-stage OSF, an increase in vasculature is an adaptive response to hypoxia and/or inflammation induced by fibrosis^6–8^. Whereas, studies have also reported that arecoline, which is the major pathogenic component in areca nuts, has a cytotoxic effect on the endothelium^9,10^. For instance, arecoline-induced autophagy can regulate apoptotic signalling in endothelial cells^11^. Arecoline can also trigger the endothelial-mesenchymal transition (EndMT) and impair endothelial cell dysfunction^12^. These findings may explain the fact that a decline in mucosal vascularity is commonly observed in late-stage OSF^12^, but the mechanisms of angiogenesis in OSF deserve further study.

Thrombospondin 1 (THBS1) is an extracellular matrix (ECM) protein that can interact with a variety of cell receptors and/or growth factors to regulate physiology and pathophysiology^13,14^. In tissue undergoing vascular remodelling, THBS1 cooperates with its receptor CD36 to counteract the angiogenic switch triggered by vascular endothelial growth factors (VEGFs)^15^. For instance, overexpression of THBS1 can suppress the survival of endothelial cells by regulating the apoptotic pathways in a CD36 dependent manner^16,17^. In multiple fibrotic diseases, THBS1 functions as a crucial activator of soluble transforming growth factor (TGF)-β1, which is a central clue that drives fibrogenesis^14,18–20^. Particularly, several studies have reported that the expression of THBS1 was upregulated in tissue with OSF^21–23^. This context raises the question of whether THBS1 can regulate angiogenesis in OSF.

Organoid technology is a powerful method to model human diseases with more physiological and clinical relevance^24–26^. In this study, the OSF and adjacent mucosa tissue was employed to generate epithelial organoids and corresponding fibroblasts. The human umbilical vein endothelial cells (HUVECs), fibroblasts and organoids were used to generate assembled organoid that can recapitulate the interplay between epithelium and mesenchyme. The clinical samples, assembled organoid, and an arecoline-induced rat model were integrated to test the regulatory role of THBS1 on vascularization in OSF.

## Results

### Overexpression of THBS1 in OSF is recapitulated in a human FAO model

**Fig. 1:**
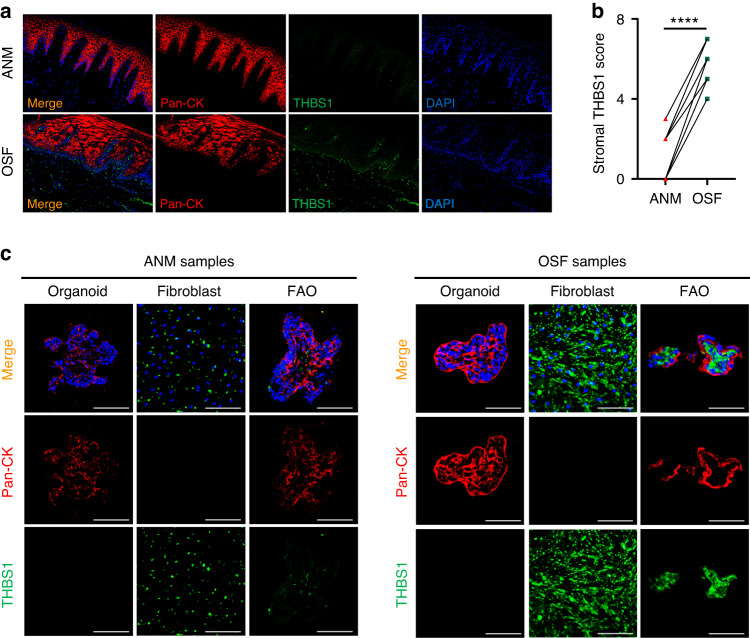
Overexpression of THBS1 in OSF is recapitulated in a human FAO model. **a** Representative images of immunofluorescence (IF) staining showed the expression pattern of THBS1 and pan-cytokeratin (pan-CK) in OSF and normal mucosa (NM) tissue. scale bars, 100 μm. **b** Quantification analysis of IF staining showed the stromal THBS1 score in OSF and normal mucosa (NM) tissue. *n* = 10. ****P < 0.000 1. **c** Representative images showed the IF staining of THBS1 and pan-CK in organoid, fibroblast, and FAO generated using OSF and normal samples, scale bars, 100 μm

To determine the expression of THBS1 in tissue with OSF, a panel of samples was collected from patients with a history of betel quid chewing history and pathological diagnoses (Table S1 and Fig. S1a). The immunofluorescence (IF) staining results confirmed that the expression of THBS1 rarely overlapped with that of epithelial cell markers, such as pan-cytokeratin (Pan-CK) (Fig. 1a). In addition, the expression level of THBS1 was increased in OSF tissue compared to the adjacent normal mucosa (ANM) (Fig. 1a, b). These results indicate that overexpression of THBS1 in stromal cells may contribute to OSF development. To better study the role of THBS1 in regulating OSF pathogenesis, epithelial organoids and corresponding fibroblasts, which are an important stromal cell type, were generated using OSF and ANM tissue. The IF and immunoblotting (IB) results validated that the expression of THBS1 was increased in fibroblasts compared to epithelial cells (Fig. 1c, Fig. S1c). Likewise, the expression of THBS1 was upregulated in OSF fibroblasts compared to ANM fibroblasts (Fig. 1c, Fig. S1c). Then, fibroblasts and organoids were used to construct a fibroblast-attached organoid (FAO) model according to our previously reported protocol^23^ (Fig. S1b). A high-content imaging analysis showed that the FAO can stably recapitulate the direct co-culture of epithelial cells and corresponding fibroblasts (Fig. S1d). Besides, the IF and IB results confirmed that the expression of THBS1 in OSF-derived FAO, which was highly restricted to the stromal compartment, was elevated compared to that in ANM-derived FAO (Fig. 1c, Fig. S1b). These results indicate that overexpression of THBS1 in OSF was recapitulated in FAOs. Consistent with these findings, treatment with transforming growth factor (TGF)-β1, a key factor that drives tissue fibrosis, promoted the expression of THBS1 in ANM fibroblasts (Fig. S2a). Furthermore, the Chromatin immunoprecipitation (ChIP)-PCR assay confirmed that TGF-β1 promoted the enrichment of p-Smad3 at the promoter of THBS1 (Fig. S2b). These results indicate that overexpression of THBS1 in fibroblasts correlated with development of OSF.

### Arecoline promotes the expression of stromal THBS1 in FAOs

**Fig. 2:**
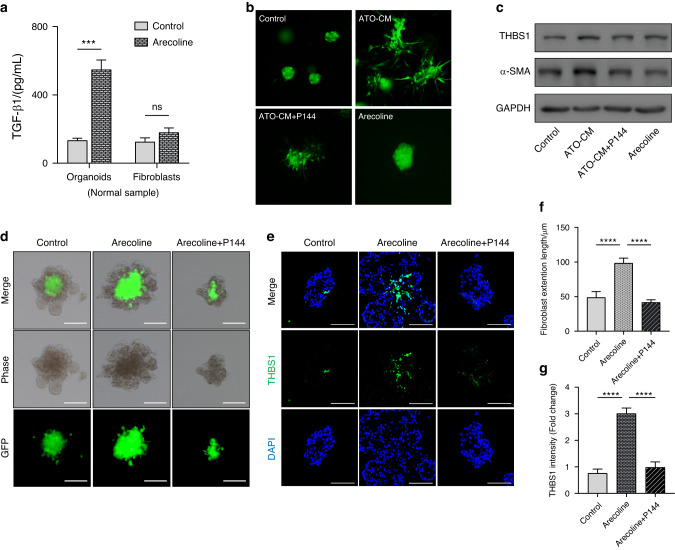
Arecoline promotes the expression of stromal THBS1 in FAO. **a** Enzyme-linked immunosorbent assay (ELISA) detected TGF-β1 concentration in epithelial organoids or fibroblasts derived conditional medium (CM) upon arecoline treatment (10 μg/mL). ***P < 0.001. **b** Representative images showed the morphology of fibroblasts cultured in the matrigel, treated with arecoline-treated organoid derived CM (ATO-CM), ATO-CM + P144 (100 μg/mL) and arecoline (10 μg/mL), scale bars, 100 μm. **c** IB assay showed the expression of THBS1 in fibroblasts treated ATO-CM, ATO-CM + P144 (100 μg/mL) and arecoline (10 μg/mL). **d** Representative images show the morphology of FAO upon treatment with arecoline (10 μg/mL) and arecoline + P144 (100 μg/mL), scale bars, 100 μm. **e** Representative IF staining images showed the expression pattern of THBS1 in FAO upon treatment with arecoline (10 μg/mL) and arecoline (10 μg/mL) + P144 (100 μg/mL), scale bars, 100 μm. **f** Fibroblast (GFP-labelled) extension length in FAO was used as a quantity index. ****P < 0.000 1. **g** Quantification analysis of IF staining showed the expression of THBS1 in FAO. ****P < 0.000 1

Studies have reported that arecoline, a major pathogenic component in betel nuts, can promote the secretion of TGF-β1 in epithelial cells^27,28^. To compare TGF-β1 secretion by cells in our model, conditioned medium (CM) from normal organoids and corresponding fibroblasts was collected for analysis. The enzyme-linked immunosorbent assay (ELISA) results showed that the concentration of TGF-β1 in organoid-derived CM was higher than that in fibroblast-derived CM (Fig. S3a). In addition, the level of TGF-β1 in organoid-derived CM was drastically increased in response to treatment with 1–100 μg/mL arecoline (Fig. 2a and Fig. S3a). To clarify whether organoid-derived TGF-β1 could trigger the phenotypic activation of fibroblasts, the CM of organoids that were treated with arecoline (ATO-CM) was used for further experiments. The colony formation assay showed that treatment with ATO-CM promoted the morphological activation of fibroblasts (Fig. 2b). In addition, the IB results confirmed that the expression of THBS1, as well as the fibroblast activation marker α-SMA, was increased in fibroblasts in response to treatment with ATO-CM (Fig. 2c). Then, disitertide (P144) diammonium, a potent antagonist of the TGF-β1 receptor^29^, was used to validate the role of TGF-β1 in ATO-CM. As confirmed by the IB results, treatment with P144 at the indicated dose reduced the expression of THBS1 in normal fibroblasts that were incubated with exogenous TGF-β1 (Fig. S3b, c). Consistent with this, ATO-CM-mediated activation of normal fibroblasts was blocked by supplementation with P144 (Fig. 2b, c), indicating that organoid-derived TGF-β1 could trigger the phenotypic activation of fibroblasts. Unexpectedly, treatment with arecoline at the indicated dose (10 μg/mL) did not affect the morphological activation or the expression of THBS1 in fibroblasts (Fig. 2b, c). To study the role of arecoline in a model that better mimics the epithelial-stromal cell interaction, FAOs were used for the arecoline treatment assay. Fibroblasts, which were labelled with GFP, were spontaneously distributed at the centre of the FAO (Fig. 2d). Treatment with arecoline promoted the migration of fibroblasts towards the periphery of the FAO (Fig. 2d, f), and this effect was blocked by P144 (Fig. 2d, f). Consistent with this, IF staining confirmed that treatment with arecoline promoted the expression of THBS1 in the FAO (Fig. 2e, g), and this effect was blocked by P144 (Fig. 2e, g). Taken together, these results indicate that arecoline can enhance the secretion of TGF-β1 by epithelial cells to promote the expression of THBS1 in fibroblasts.

### Fibroblast-derived THBS1 suppresses the sprouting ability of endothelial cells

**Fig. 3:**
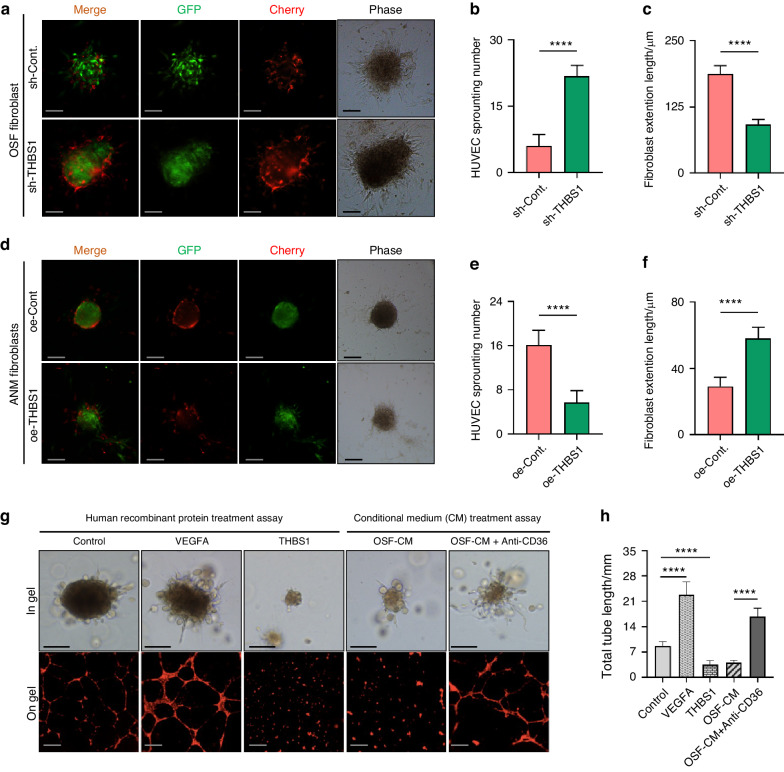
Fibroblast-derived THBS1 suppresses the sprouting ability of endothelial cells. **a**–**f** Representative images and quantitative analysis showed the mixture of fibroblasts and human umbilical vein endothelial cells (HUVECs) in the fibroblast-HUVEC clusters. Quantification analysis validated that knockdown of THBS1 in fibroblasts in OSF (OSF fibroblast) increased the sprouting number of HUVECs (**b**) but decreased the length of fibroblasts (**c**). Overexpression of THBS1 in normal fibroblasts (ANM fibroblast) reduced the sprouting number of HUVECs (**d**, **e**) but induced the length of fibroblasts (**d**, **f**), scale bars, 100 μm. ****P < 0.000 1. **g** Representative images of HUVEC colony in gel and the tube formation on gel, treated with vascular endothelial growth factor A (VEGFA, 1 μg/mL), THBS1 (1 μg/mL), OSF-fibroblast derived CM (OSF-CM) and OSF-CM + Anti-CD36, scale bars, 100 μm. **h** Quantitative analysis of the length of tubes formed by HUVECs. ****P < 0.000 1

Studies have reported that THBS1 can interact with its receptor CD36 to suppress angiogenesis^17^. To clarify the role of THBS1 in regulating angiogenesis during OSF development, fibroblasts generated from OSF or ANM tissue were used for analysis. First, OSF fibroblasts were transfected with a short-hairpin RNA targeting the expression of THBS1 (sh-THBS1). Reductions in fibroblast expression and secretion of THBS1 were confirmed by IB and ELISA (Fig. S4a, b). Then, the fibroblasts were cocultured with human umbilical vein endothelial cells (HUVECs) at a 3:1 ratio using a cell cluster culture approach^26^. A high-content imaging analysis^30^ confirmed that the sprouting of EC was recapitulated in this co-culture model (Fig. S4e, f). Likewise, knockdown of THBS1 in OSF fibroblasts promoted the sprouting of ECs (Fig. 3a–c). On the other hand, THBS1 was overexpressed in ANM fibroblasts (Fig. S4c, d). In the same coculture model, overexpression of THBS1 in fibroblasts impaired the sprouting of ECs (Fig. 3d–f). To further validate the role of fibroblast-derived THBS1 in regulating endothelial cell proliferation, human recombinant THBS1 (hrTHBS1) and the conditioned medium of OSF fibroblasts were collected for HUVEC treatment assays. As confirmed by in gel sprouting and on gel tube formation assays, treatment with hrTHBS1 or fibroblast-derived CM reduced the growth of ECs (Fig. 3g, h). The suppressive effects of fibroblast-derived CM were attenuated when the HUVECs were incubated with anti-CD36 antibodies (Fig. 3g, h). Since the interaction of THBS1 with CD36 can activate the intrinsic and extrinsic apoptotic pathways in endothelial cells (ref), to verify the angiostatic role of THBS1 in our model, the expression of effector caspase, such as caspase-3 in HUVEC was also analysed. Treatment with fibroblast-derived CM promoted the expression of caspase-3 in HUVEC, and this effect was rescued by supplement with anti-CD36 antibodies (Fig. S4g). Together, these results indicate that fibroblast-derived THBS1 can suppress the growth dynamics of endothelial cells in a CD36-dependent manner.

### Arecoline reduces the expression of endothelial marker in vascularized FAOs

**Fig. 4:**
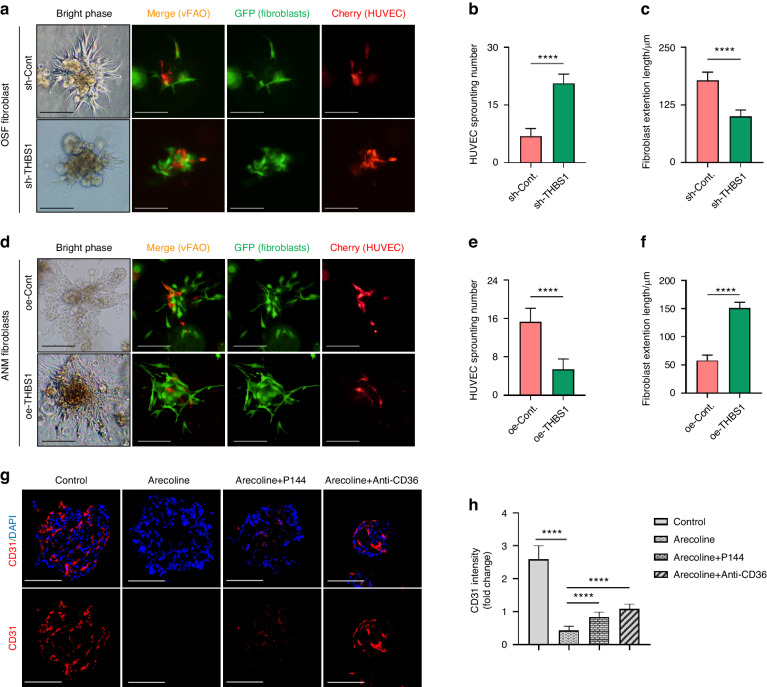
Arecoline reduces the expression of endothelial marker in vascularized FAOs. **a**–**f** Representative images showed the distribution and morphology of fibroblasts (GFP-labeled) and HUVECs (mCherry-labeled) in the vascularized FAO (v-FAO). Quantification analysis validated that knockdown of THBS1 in fibroblasts in OSF (OSF fibroblast) increased the sprouting number of HUVECs (**b**) but decreased the extension length of fibroblasts (**c**). Overexpression of THBS1 in adjacent normal fibroblasts (ANM fibroblast) reduced the sprouting number of HUVECs (**e**) but induced the extension length of fibroblasts (**f**), scale bars, 100 μm. ****P < 0.000 1. **g** Representative images of IF staining showed the expression of CD31 in v-FAO treated with arecoline (10 μg/mL), arecoline (10 μg/mL) + P144 (100 μg/mL) and arecoline (10 μg/mL) + Anti-CD36. **h** Quantification analysis of CD31 expression in v-FAO. ****P < 0.000 1

To study the role of THBS1 in a model that better simulates the perivascular niche, HUVECs were incorporated into FAOs to mimic the vascular component (see Materials and Methods for more detail). As shown in Fig. 4a–c, in vascularized FAOs (vFAOs) that were generated using OSF tissue, knockdown of THBS1 in fibroblasts not only inhibited their morphological activation (Fig. 4a, b) but also enhanced the sprouting of HUVECs (Fig. 4a, c). On the other hand, in vFAOs that were generated using ANM tissue, HUVEC sprouting was decreased when THBS1 was overexpressed in fibroblasts (Fig. 4d–f). In line with this, treatment with arecoline, which modelled the pathogenic factor of OSF (ref), reduced the expression of CD31, a widely used endothelial marker (ref) in vFAOs (Fig. 4g, h). To clarify the mechanism by which arecoline affect CD31 expression, P144 were supplemented to block the TGF-β1 secretion in epithelial cells, which is the upstream signal of THBS1 in our model (as mentioned above). As a results, P144 rescued the arecoline-induced reduction in CD31 expression (Fig. 4g, h). Furthermore, supplementation of anti-CD36 to block the interaction of THBS1 with CD36 also declined the arecoline-induced reduction in CD31 expression (Fig. 4g, h). Taken together, these results indicated that arecoline can hijack THBS1 in fibroblasts to suppress the growth dynamics of endothelial cells.

### Inhibiting THBS1 alleviates disease progression in a rat OSF model

**Fig. 5:**
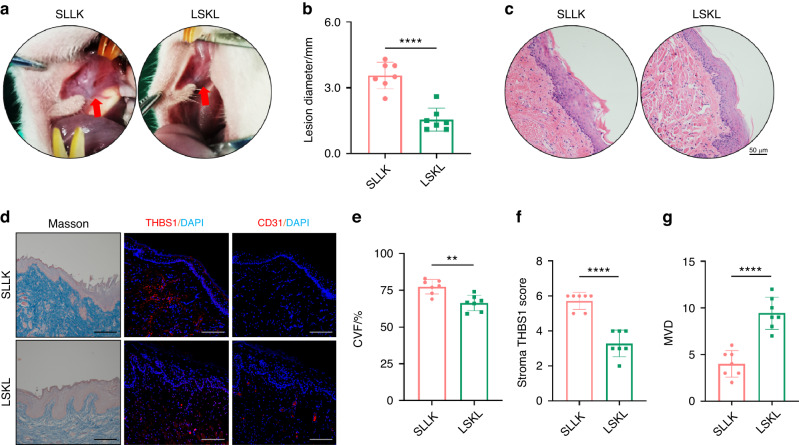
Inhibiting THBS1 alleviates disease progression in a rat OSF model. **a** Representative image showed the white patch in oral mucosa of SD rats treated with SLLK or LSKL. **b** Lesion diameter was measured and quantitative analysis showed that it was much longer in SLLK group compared than that in LSKL group. *n* = 7. ****P < 0.000 1. **c** HE staining confirmed histological change of specimens in SLLK group and LSKL group, scale bars, 50 μm. **d** Representative images of Masson staining and IF staining showed the collagen deposition and the expression of THBS1 and CD31 in each group, scale bars, 100 μm. **e**–**g** quantitative analysis of collagen volume fraction (CVF), the stromal score of THBS1 and the microvessel density (MVD) in SLLK group and LSKL group. **P < 0.01. ****P < 0.000 1

To investigate the targeting value of THBS1, an in vivo rat OSF model was generated. Briefly, 28 SD rats were randomly allocated into four groups (i.e., A, B, C, D). DMSO (solvent of arecoline) and arecoline were injected into the buccal mucosa of rats in Groups A (Control) and B (Arecoline), respectively. Mouth opening was measured once per week. The masticatory movement of rats in the arecoline group gradually decreased (Fig. S5a). However, there were no alterations in mouth opening in the control group (Fig. S5a). Once the mucosa became blanched, specimens with lesions were harvested (Fig. S5b, c). Masson staining confirmed that the expression of collagen was drastically increased in the arecoline group (Fig. S5d, e), indicating that treatment with arecoline could induce the pathological alterations of OSF in rats. Consistently, THBS1 overexpression and a reduction in CD31 were observed in rat OSF tissues (Fig. S5d, f, g). These findings indicated that THBS1 overexpression correlated with a decrease in vascularity during OSF development. Based on this modelling approach, the inhibitor of THBS1 (LSKL) and its control peptide (SLLK)^31^ were administered to rats in Group C and D. The diameter of white mucosal patches was recorded (Fig. 5a, b). At week 12, the specimens were harvested for analysis (Fig. 5c). Masson and IF staining showed that the expression of collagen and THBS1 was reduced in rats that received LSKL treatment (Fig. 5d–f). More interestingly, the expression of CD31 was drastically increased in response to treatment with LSKL (Fig. 5d, g). In conclusion, these results indicated that targeting THBS1 could alleviate the development of OSF.

## Discussion

**Fig. 6:**
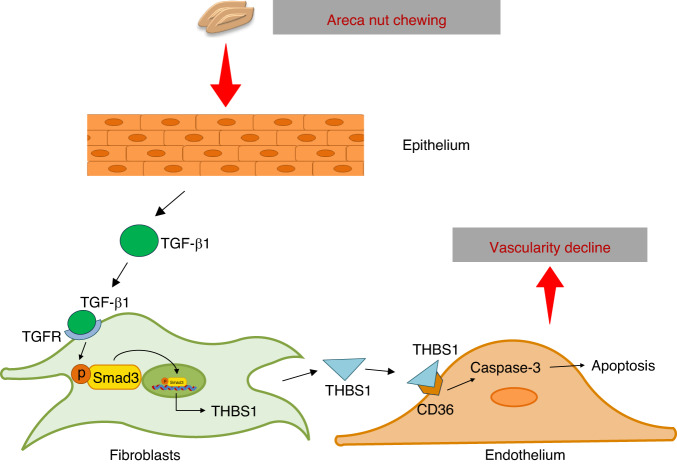
Schematic of the proposed mechanism by which THBS1 suppresses angiogenesis in OSF

Current studies of OSF pathogenesis are largely limited by the available models. Here, the organoid-stromal cell coculture model (assembled organoids) was used to investigate the role of THBS1 in regulating OSF development. THBS1 overexpression in OSF, which is highly restricted to stromal cells, was stably recapitulated in fibroblast-attached organoids (FAOs). In pathogenic factor stimulation experiments, arecoline enhanced the secretion of TGF-β1 by epithelial cells, which then promoted the expression of THBS1 in fibroblasts. Furthermore, HUVECs were incorporated into FAOs to mimic the vascular component. Overexpression of THBS1 in fibroblasts suppressed the growth dynamics of endothelial cells in a CD36-dependent manner (Fig. 6). The targeting value of THBS1 was further validated in an arecoline-induced rat OSF model. Overall, we used an assembled organoid model to study OSF pathogenesis and suggest that THBS1 is a promising therapeutic target.

Arecoline is a major pathogenic component in areca nuts. Many studies have reported that arecoline can trigger the phenotypic transition of epithelial cells in the oral mucosa^32–34^. Recently, it was suggested that arecoline could affect the cell‒cell interplay in the OSF environment^34^. However, the limitations of the available models have challenged the validation of this hypothesis. Here, epithelial cells and corresponding fibroblasts were generated from oral mucosa tissues. In an individual culture model, treatment with arecoline promoted the secretion of TGF-β1 by epithelial organoids. However, treatment with arecoline at a similar dose did not affect the morphological activation of fibroblasts or the expression of activation markers in fibroblasts. This lack of effects of arecoline on fibroblasts are consistent with other previous studies^27^. This phenomenon may be due to the inability of individual cultures to model cellular crosstalk in the OSF environment. To overcome this issue, we constructed a direct coculture model of epithelial cells and fibroblasts (FAOs), according to our previously reported protocol^26^. In the FAO, treatment with arecoline at a similar dose induced the morphological activation of fibroblasts. These findings indicated that the assembled organoid model could be used to study how arecoline affects cellular interactions in the OSF environment.

Disturbances in TGF-β signalling are implicated in most, if not all, human fibrotic diseases^35,36^. Of the TGF-β isoforms, TGF-β1 can directly promote the proliferation, migration and survival of fibroblast-type cells^37^. Mechanistically, TGF-β1 cascades affect Smad proteins to activate the transcription of profibrotic molecules, including collagens and fibronectin^38^. In an animal model, deletion of the gene encoding the receptor of TGF-β drastically reduced the production of collagen I^39^. These results emphasize the potential of intervening in TGF-β1-related pathways to alleviate fibrosis. Although fibroblasts are considered to be the main cells that respond to TGF-β1, the cells that produce TGF-β1 can be largely context dependent. In the OSF environment, it has been shown that arecoline can trigger the secretion of TGF-β1 by epithelial cells^27,28^. Here, we compared the secretion of TGF-β1 by epithelial cells and corresponding fibroblasts generated from oral mucosa tissue. We found that epithelial cells could secrete more TGF-β1 than fibroblasts. More importantly, treatment with epithelial cell-derived CM promoted the activation of fibroblasts in a TGF-β1-dependent manner. These findings indicate that arecoline can hijack epithelial cell-derived TGF-β1 to promote OSF development.

THBS1 is a prototypical ECM protein that orchestrates cell-matrix interactions. On the one hand, THBS1 can act as a protease to cleave the precursor form of TGF-β (latent TGF-β), which is surrounded by the latency-associated peptide (LAP)^18^. Knockout of *THBS1* in mice reduced the abundance of the active form of the TGF-β protein^40^. This evidence suggests that THBS1 overexpression can contribute to ECM remodelling by activating soluble TGF-β. On the other hand, THBS1 expression appears to be regulated by TGF-β signalling^20^. In a study of glioblastoma, RNA-seq analysis revealed that *THBS1* was involved in the target genes of the Smad protein^20^, indicating that TGF-β and THBS1 can cooperate to form a positive feedback loop. Here, we focused on the expression and function of THBS1 in OSF tissues. Using an FAO model, THBS1 overexpression in OSF, which is highly restricted to the stromal compartment, was stably recapitulated in vitro. In the FAO, treatment with arecoline promoted the expression of THBS1 in a TGF-β1-dependent manner. In an arecoline-induced rat OSF model, inhibition of THBS1 alleviated collagen deposition and the decline in vascularity. Taken together, these findings suggest that THBS1 overexpression can contribute to the development of OSF.

In patients with middle- and late-stage OSF, the oral mucosa always becomes blanched due to the reduction in blood supply^8^. However, the investigation of angiogenesis in OSF is still in its infancy. In a pioneering study, Tseng et al. showed that arecoline could induce cell cycle arrest in endothelial cells and thereby regulate vascular dysfunction^9^. More recently, Li et al. showed that the ROS/PERK/YAP pathway was triggered by arecoline in vitro and in vivo, which could lead to the endothelial-mesenchymal transition in OSF tissue^12^. In this study, we found that the expression of THBS1 was increased in OSF tissue. Overexpression of THBS1 could inhibit the survival of endothelial cells by triggering the CD36/p38 signalling axis^16^. Therefore, we examined whether THBS1 could regulate microvascular changes in OSF tissue. We constructed fibroblast-endothelial cells co-culture and vascularized FAO (vFAO) models using HUVECs, which are a widely used source of endothelial cells. Overexpression of THBS1 in fibroblasts reduced the sprouting of endothelial cells in both of the coculture models. Importantly, fibroblast-derived CM enhanced the sprouting of endothelial cells in a CD36-dependent manner. In vFAO models, arecoline reduced the expression of CD31, and these effects were ameliorated when the endothelial cells were preincubated with anti-CD36 antibodies. Overall, these findings indicate that stromal THBS1 can suppress angiogenesis in OSF tissue.

Our study has limitations. First, the number of clinical samples was relatively small. It is essential to clarify the expression pattern of THBS1, as well as its correlation with microvascular density in more human OSF samples. Regarding to the experiment model, although FAO can recapitulate the direct co-culture of epithelial cell and fibroblast, the spatial regulation of cells in FAO deserved further investigated. Future studies based on single cell sequencing and high-content imaging system may help to figure out this issue. Regarding to the mechanism investigation, it should be noticed that fibroblasts may influence endothelial cell proliferation through various pathways^41,42^. Although we suggest that THBS1/CD36 signal axis was involved in the arecoline-induced fibroblasts activation and endothelial cells dysfunction, more unbiased researches are needed to clarify how stromal cells affect perivascular niche in similar context. However, the use of assembled organoid provides a novel strategy to study the OSF pathogenesis with more clinical and physiological relevance. We believe that such model may also benefit to the development of therapeutic target for patients with OSF.

## Materials and methods

### Collection of clinical samples

OSF and their adjacent normal mucosa (ANM) tissues were collected from the surgical specimens of OSCC patients with betel nut chewing history (*n* = 10), according to IRB–approved guidelines at the Ethics Committee of School and Hospital of Stomatology at Wuhan University (IRB-ID:2021A18). Clinical information of relevant samples has been described in supplementary table 1. The pathological diagnosis of OSF samples was processed by 2 experienced pathologists according to the Pindborg and Sirsat criteria: excessive collagen deposition in the connective tissues below the oral mucosal epithelium, local inflammation in the lamina propria or deep connective tissues, obliterated vessels and degenerative changes in the muscles^2,43^.

### Organoid culture

OSF and ANM tissues were minced into small pieces and dissociated into single cells for organoid culture, the composition of basal organoid culture medium was prepared as we recently described^44,45^. Advanced DMEM/F12 was supplemented with 1 x N2 (07152, Stemcell technology, CA), 1 x B27 (7511, Stemcell technology, CA), 10 μmol/L Y-27632 (HY-10071, MCE, USA), 0.5 μmol/L A83-01 (HY-10432, MCE, USA), 10 μmol/L forskolin (HY-15371, MCE, USA) and 10 ng/mL EGF (236-EG-200, R&D, USA)^44,45^. For organoid passage assay, 125 ng/mL Noggin (6057, R&D system, USA), 125 ng/mL R-spondin (4645, R&D system, USA) and 2 μg/mL Wnt3A (5036, R&D system, USA) were added to the basal medium. The growth dynamic of organoid was recorded under a fluorescence microscope (Leica Microsystems, GER).

### Fibroblasts and endothelial cells culture

OSF and ANM tissues were minced into small pieces for explant culture as described previously^46,47^. Briefly, the explants were digested with 0.25% trypsin containing EDTA (25200-056, Gibco, USA) for 30 min. After centrifugation, the supernatant was removed and the digested explants were then seeded to a T25 culture flask. The flask was inverted overnight at 37°C in a humidified atmosphere containing 5% CO_2_. After the explants were attached to the culture flask, MEM ALPHA (C12571500BT, Gibco, USA) containing 10% foetal bovine serum (FBS, 10099141, Gibco, USA) were added to the flask for further culture and expansion. The human umbilical vein endothelial cells (HUVECs) cell line was purchased from Sciencell (DFSC-EC-01, Sciencell, USA) and were cultured in Endothelial Cell Medium (ECM; 1001, Sciencell, USA). Cells were cultured at 37°C in a humidified atmosphere containing 5% CO_2_.

### Construction of Fibroblast-attached Organoid (FAO)

Briefly, 10^5^ epithelial cells and 3 × 10^5^ fibroblast were suspended in ultralow-attached plates (ULA) for 24–48 h to generate cell clusters, which were then embedded in Matrigel (356237, Corning, USA) to construct FAO, as we recently described^26^. To promote the efficiency of generating OSF- and ANM-derived FAO, the basal organoid culture medium was supplemented with 125 ng/mL Noggin (6057, R&D system, USA) and 125 ng/mL R-spondin (4645, R&D system, USA). The morphology and growth dynamic of FAOs was recorded under a fluorescence microscope (Leica Microsystems, GER). To validate the spatial location of epithelial cells and fibroblasts in FAOs, the cells were respectively labelled with mCherry or GFP. Then, an automated microlens-enhanced spinning disc confocal microscope (Opera Phenix^TM^ High Content Screening System, PerkinElmer) was utilized to capture the fluorescence signal of cells at distinct plane^30^. The reconstruction of superimposed images for FAOs was processed by the Harmony software v.4.9 (PerkinElmer) according to the manufacture’s instruction.

### Fibroblast-colony formation assay

Single fibroblast was suspended in ultralow-attached plate (3471, Corning) for 24 h to generate clusters. Then, the clusters were seeded in matrigel (356237, Corning, USA). Morphological characteristics were observed under a fluorescence microscope (Leica Microsystems, GER). The extension length of the cluster was measured to evaluate the fibroblast activation as described previously^48^.

### Co-culture of fibroblast and HUVEC

Single fibroblast (labeled with GFP) and HUVEC (labeled with mCherry) were mixed at a 3:1 ratio and suspended in ultralow-attached plates (ULA) for 24 h to generate fibroblast-HUVEC clusters. The cluster with diameter >40 μm was selected for embedding in matrigel (356237, Corning, USA), and the morphology of clusters was recorded under an inverted microscope (Leica Microsystems, GER). The number of cherry-labeled cells with sprouting >10 μm were counted to evaluate the activation of HUVEC. The length of GFF-labeled cells with extension >40 μm were measured to evaluate the activation of fibroblast. The sprouting of HUVEC in the co-culture model was analyzed by Opera Phenix^TM^ High Content Screening System.

### Generation of vascularized FAO (v-FAO)

Briefly, 10^5^ epithelial cells and 3 × 10^5^ fibroblast and 10^5^ HUVEC were suspended in ultralow-attached plates (ULA) for 24 h to generate cell clusters, which were then embedded in matrigel (356237, Corning, USA) to generate v-FAO. To promote the efficiency of generating v-FAO, the basal organoid culture medium was supplemented with 33.3% MEM ALPHA (C12571500BT, Gibco, USA) and 33.3% EGM-2 BulletKit (CC-3162, Lonza, CH). To analysis the phenotype transition of cells, the fibroblasts were labeled with GFP, while the HUVEC were labeled with mCherry. The growth dynamic of v-FAO, morphological activation of fibroblasts, and sprouting of HUVEC was recored using an inverted microscope (Leica Microsystems, GER).

### Immunofluorescence assay

Serial freezing section of FAO and v-FAO was processed as we recently described^44,49^. The freezing section at the core of FAO/v-FAO was employed to analysis the cell composite and protein expression. The following primary antibodies were used for immunofluorescence: THBS1 (ab1823, Abcam, UK), CD31 (3528, CST, USA), Collagen III (ab184993, Abcam, UK), Pan-Keratin (4545, CST, USA), COL1A1 (72026, CST, USA), COL3A1 (66887, CST, USA).

### Immunoblotting assay

The following primary antibodies were used for immunoblotting according to our previously reported protocol: THBS1 (18204-1-AP, Proteintech, China), Pan-Keratin (4545 S, CST, USA), Smad2 (5339, CST, USA), Phospho-Smad2 (p-Smad2, 3108, CST, USA), Smad3 (9523, CST, USA), Phospho-Smad3 (p-Smad3, 9520, CST, USA) α-SMA (19245 S, CST, USA), caspase-3, cleaved caspase-3 (ab32042, Abcam, UK).

### Chromatin immunoprecipitation (ChIP) assay

The ChIP assay was conducted according to the manufacturer’s instruction of the ChIP kit (Thermo Fisher Scientific, USA). Briefly, 2×10^6^ cells in each group were harvest for the assay. The protein-DNA complexes were cross-linked by 1% formaldehyde then quenched by glycine, after which the cells were lysed in lysis buffer. Then the lysates containing chromatin were sonicated to shear DNA. The supernatant after centrifugation was diluted in IP dilution buffer for immunoprecipitation. After incubated with antibodies or IgG at 4 °C overnight, ChIP Grade Protein A/G Magnetic Beads were used to bind chromatin DNA. The cross-links were reversed after the immunoprecipitation complexes were eluted, and then the DNA was purified and subjected for qPCR assay. The primers sequence used in the qPCR assay were listed in Table S2.

### Masson staining

Tissues of SD rats were embedded with paraffin and then mounted on slides. Masson staining was employed to evaluate the collagen deposition according to the he manufacturer’s instructions (G1006, Servicebio, China).

### Enzyme-linked immunosorbent assay (ELISA)

To measure TGF-β1 released by organoids or corresponding fibroblasts, cells were seeded into 6-well plates and stimulated with arecoline at the indicated concentrations (10 μg/mL) or not for 48 h. Then, the supernatant was harvested and the concentrations of TGF-β1 were measured using a human TGF-β1 ELISA kit (EHC107b.96, Neo Bioscience Technology, China) according to the manufacturer’s instructions. To confirm the THBS1 secretion of fibroblast with THBS1 knockdown or overexpression, the supernatant was harvested and the concentrations of THBS1 were measured using a Human THBS1/TSP1 ELISA Kit (EK0899, Raybiotech, USA).

### Tube formation assay

HUVECs were cultured in Endothelial Cell Medium (1001, Sciencell, USA). When reached 80% confluence, the HUVECs were seeded onto 96 well plates embedded with matrigel in the presence of VEGFA, hrTHBS1, fibroblast CM and Anti-CD36, respectively. Tube formation was visualized under inverted microscope (Leica Microsystems, GER) at 12 h after incubation. Total tube length was measured for analysis.

### Animal experiments

For animal experiments, 28 female Sprague-Dawley (SD) rats were purchased from Beijing Vital River Laboratory Animal Technology Co., Ltd. and maintained according to the protocol approved by the Ethics Committee of the Hospital of Stomatology at Wuhan University (S07921060J). Sprague-Dawley (SD) rats were purchased from Beijing Vital River Laboratory Animal Technology Co., Ltd. In total, 28 SD rats were randomly divided into four groups (i.e. A, B, C, D). The submucosal injection method on the right cheek was used in this experiment. Arecoline (S2614, Selleck, USA), LSKL (HY-P0299, MCE, USA) and SLLK (HY-P0301, MCE, USA) were dissolved in DMSO (A3672, Applichem, GER) to 10 mg/mL for storage and were diluted to 2 mg/mL in 0.9% normal saline (NS) immediately before injection. To establish the OSF lesion (*n* = 7), the rats in group A were injected with 20% DMSO and the rats in group B were injected with arecoline. As for drug administration assay (*n* = 7), the rats in group C were injected with SLLK and the rats in group D were injected with LSKL. All groups were injected twice a week and with 100 μL each time. The mouth opening of animals were measured once a week. After 12 weeks, the rats were humanely euthanized, and the buccal tissue was attained for further research.

## Statistical analysis

All experiments were performed in triplicate and repeated at least 3 times. Data were analyzed using SPSS v.16.0 (SPSS, Chicago, IL, USA) and Prism v.5.0 (GraphPad Software, La Jolla, CA, USA). The results were expressed as means + SD. Differences with values of *P* < 0.05 were considered significant.

### Supplementary information

Revised supplementary information
