# Supplementary material for: Stromal thrombospondin 1 suppresses angiogenesis in oral submucous fibrosis
Source: Int J Oral Sci. 2024 Feb 26;16:17. doi: 10.1038/s41368-024-00286-z (PMC10894862; doi:10.1038/s41368-024-00286-z)
Supplement: Supplementary file 1 — Revised supplementary information [file 41368_2024_286_MOESM1_ESM.docx]

**Table S1. Clinical feature of patients with OSF.**


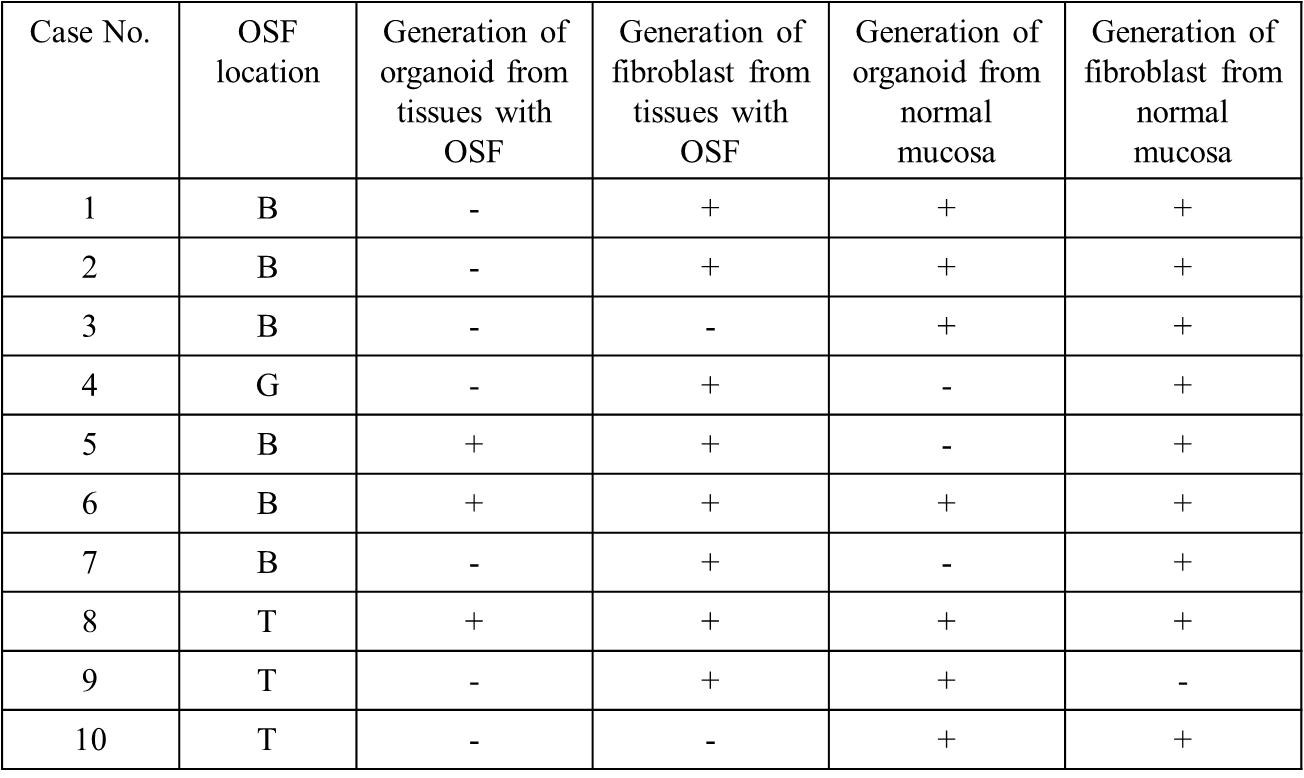


Abbreviations: T-Tongue, B-Buccal mucosa, G-Gingiva. “+” indicated success, “-” indicated failure.

**Table S2. Primer sequence for qPCR**

| Gene | Forward primer | Reversed primer |
| --- | --- | --- |
| *GAPDH* | ACAACTTTGGTATCGTGGAAGG | GCCATCACGCCACAGTTTC |
| *THBS1* | GCCATCCGCACTAACTACATT | TCCGTTGTGATAGCATAGGGG |

**Figure S1**

**
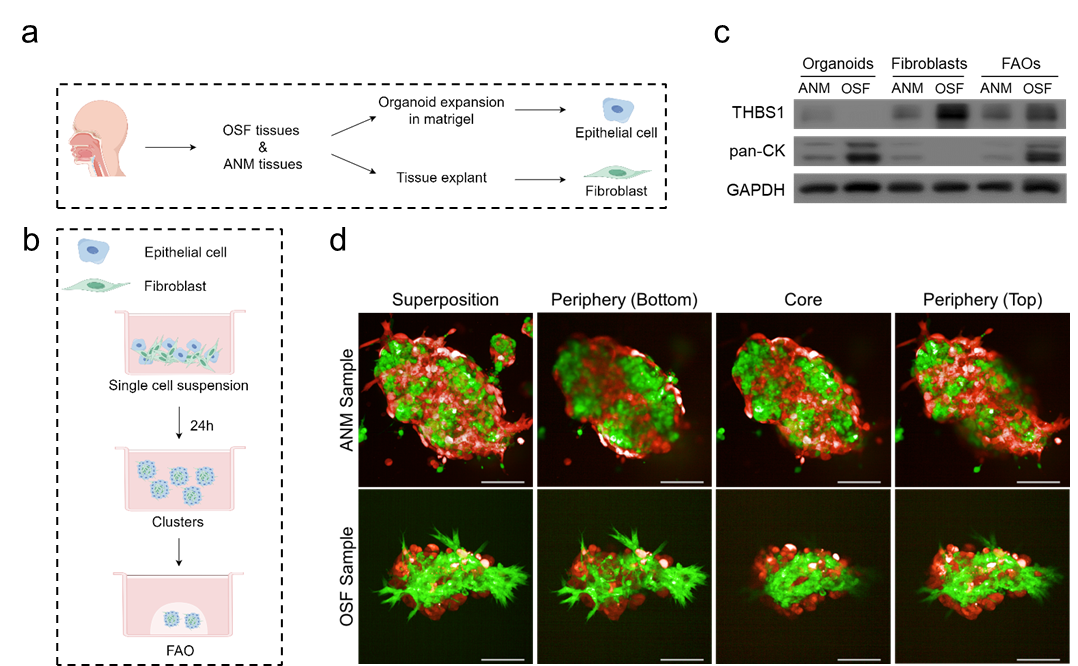
**

Figure S1. Generation of the fibroblast attached organoid (FAO). **a** Generation of organoid and the corresponding fibroblast from OSF tissue and the adjacent normal mucosa (ANM). **b** Schematic of the key steps for generating the fibroblast attached organoid (FAO). **c** Immunoblotting (IB) assays of THBS1 and pan-CK in organoids, fibroblasts and FAOs generated from ANM and OSF tissues. **d** High-content imaging analysis of FAO. The schematic were constructed by Figdraw.

**Figure S2**


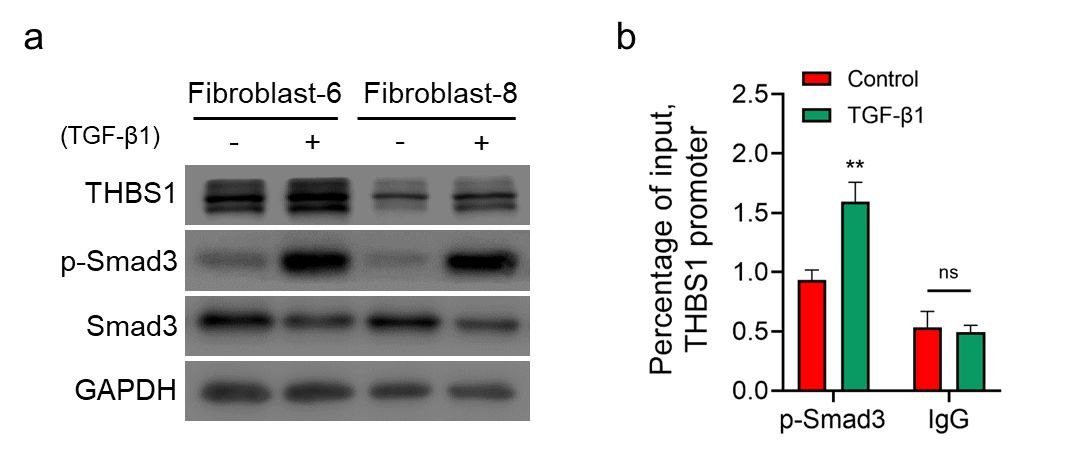


Figure S2. TGF-β1 promotes the transcriptional activation of THBS1 via Smad3/ phosphorylated (p-Smad3). **a** Result of IB assay showed that treatment with TGF-β1 increased the ratio of p-Smad3/Smad3 as well as the expression of THBS1. **b** The result of ChIP-PCR showed that treatment with TGF-β1 induced the binding of p-Smad3 to the promoter of *THBS1* gene.

**Figure S3**


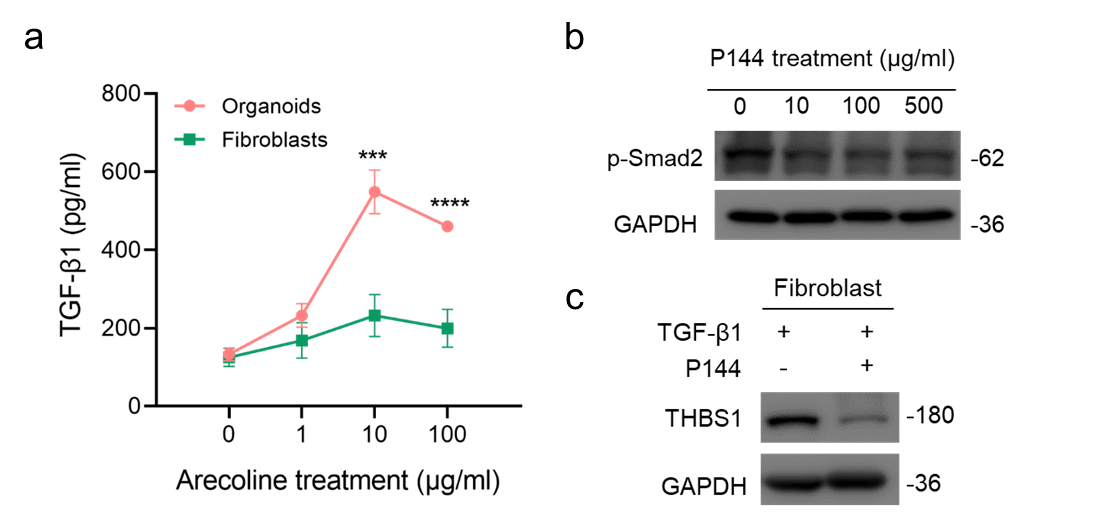


Figure S3. Organoid-derived TGF-β1 up-regulated the expression of THBS1 in fibroblasts. **a** Concentration curve of TGF-β1 in conditional medium of organoid and fibroblast that were incubated with arecoline at dosage of 0-100 μg/ml. ***P<0.001, ****P<0.0001. **b** Treatment of P144 reduced the phosphorylation of Smad2 in fibroblast incubate with TGF-β1 at 10ng/ml. **c** TGF-β1-mediated overexpression of THBS1 was reduced by treatment with P144 at 100 μg/ml.

**Figure S4**


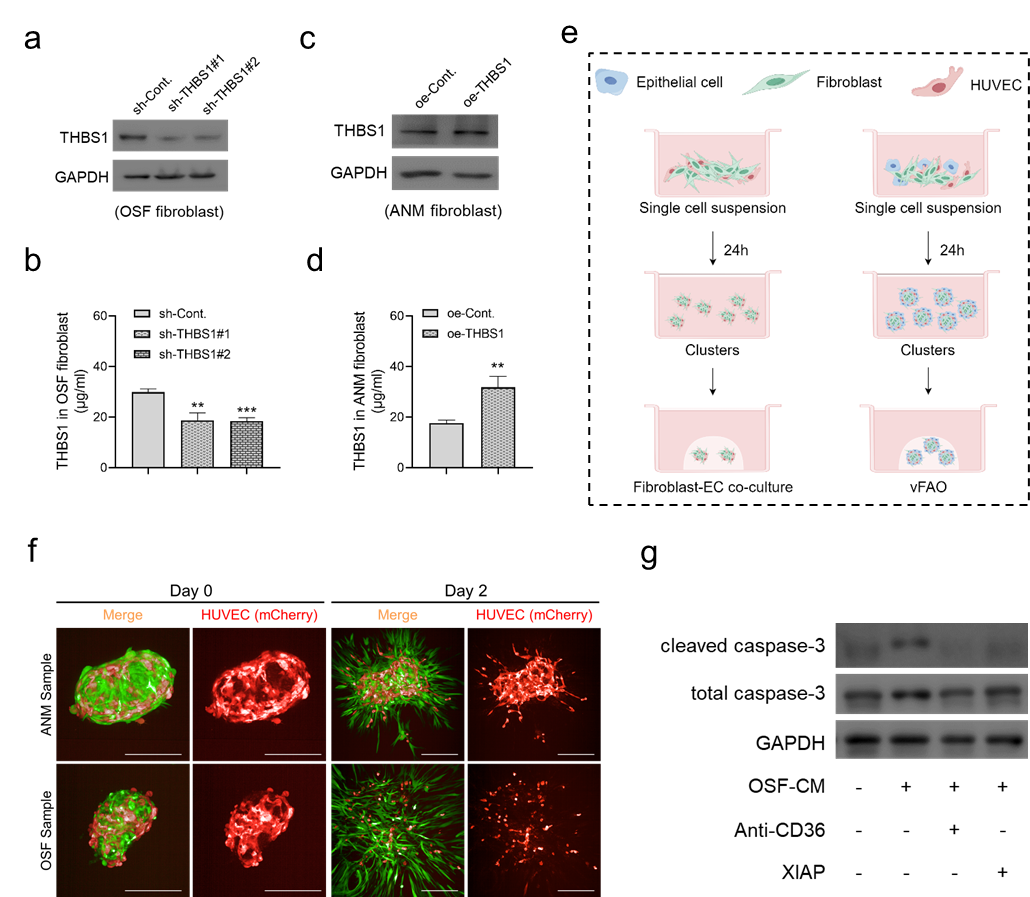


Figure S4. Fibroblast-derived THBS1 suppress the growth dynamics of endothelial cells. **a, b** IB assay (a) and ELISA (b) confirmed decreased expression and secretion of THBS1 in OSF derived fibroblasts (OSF fibroblast) that were transfected with a short hairpin RNA toward human THBS1 (sh-THBS1). **P < 0.01. ***P < 0.001. **c, d** IB assay (c) and ELISA (d) confirmed increased expression and secretion of THBS1 in adjacent normal fibroblasts (ANM fibroblast) that were transfected overexpression lentiviral vector of THBS1 (oe-THBS1). **P < 0.01. **e** Schematic of the key steps for generating the fibroblast-HUVEC (fibroblast-EC) co-culture model and vascularized FAO (vFAO). **f** High-content imaging analysis of fibroblast-HUVEC co-culture model. **g** IB assay of cleaved caspase-3 expression in HUVECs treated with fibroblast-derived conditional medium (CM), anti-CD36 antibody and X-linked inhibitor of apoptosis protein (XIAP, an inhibitor of apoptosis). The schematic were constructed by Figdraw.

**Figure S5**


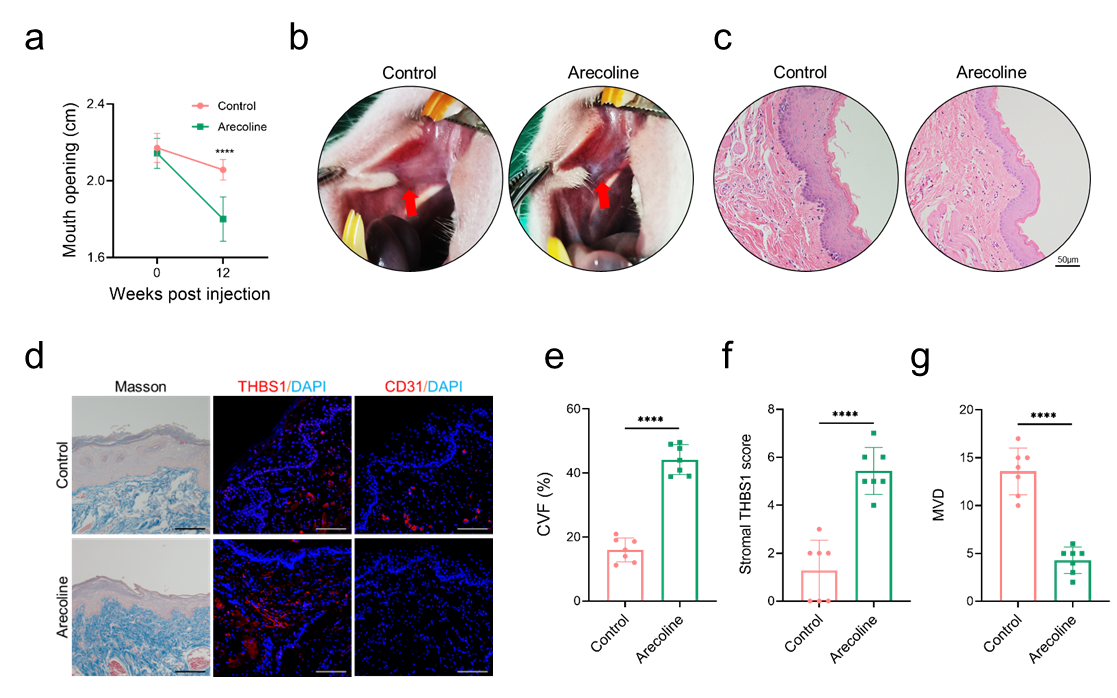


Figure S5. Arecoline induce the pathological alterations of OSF in Sprague-Dawley (SD) rats. **a** Mouth opening of rat in each group were measured. Quantitative analysis showed that the mouth opening was markedly decreased in the arecoline group, but no alternation was found in control group. n = 7. ****P < 0.0001. **b** Representative images showed the white patch in oral mucosa of rats. **c** HE staining confirmed histological change in rat OSF specimens. scale bars, 50μm. **d** Representative images of Masson staining and IF staining showed the collagen deposition and the expression of THBS1 and CD31 in each group, scale bars, 100μm. **e ~ g** Quantitative analysis of collagen volume fraction (CVF), the stromal score of THBS1 and the microvessel density (MVD). ****P < 0.0001.
